# Immune‐relatedlncRNAs can predict the prognosis of acute myeloid leukemia

**DOI:** 10.1002/cam4.4487

**Published:** 2021-12-14

**Authors:** Ran Li, Shishuang Wu, Xiaolu Wu, Ping Zhao, Jingyi Li, Kai Xue, Junmin Li

**Affiliations:** ^1^ Shanghai Institute of Hematology State Key Laboratory of Medical Genomics National Research Center for Translational Medicine at Shanghai Ruijin Hospital Affiliated to Shanghai Jiao Tong University School of Medicine Shanghai China; ^2^ Department of Children Health Care Women's Hospital of Nanjing Medical University Nanjing Maternity and Child Health Care Hospital Nanjing China; ^3^ Department of Biology University of North Alabama Florence Alabama USA

**Keywords:** acute myeloid leukemia, immune‐related lncRNAs, nomogram, prognosis, tumor microenvironment

## Abstract

The immune microenvironment in acute myeloid leukemia (AML) is closely related to patients’ prognosis. Long noncoding RNAs (lncRNAs) are emerging as key regulators in immune systems. In this study, we established a prognostic model using an immune‐related lncRNA (IRL) signature to predict AML patients’ overall survival (OS) through Least Absolute Shrinkage and Selection Operator (LASSO) and multivariate Cox regression analysis. Kaplan‐Meier analysis, receiver operating characteristic (ROC) analysis, univariate Cox regression, and multivariate Cox regression analyses further illustrated the reliability of our prognostic model. An IRL signature‐based nomogram consisting of other clinical features efficiently predicted the OS of AML patients. The incorporation of the IRL signature improved the ELN2017 risk stratification system's prognostic accuracy. In addition, we found that monocytes and metabolism‐related pathways may play a role in AML progression. Overall, the IRL signature appears as a novel effective model for evaluating the OS of AML patients and may be implemented to contribute to the prolonged OS in AML patients.

## Graphical abstract

The immune‐related lncRNA signature represents a novel effective model for evaluating AML survival outcomes and may be implemented to contribute to the prolonged OS in AML patients.

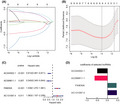

## 1 INTRODUCTION

Acute myeloid leukemia (AML) is the most prevalent leukemia in adults with a sequence of somatic mutations that cause the abnormal growth and differentiation of hematopoietic stem cells (HSCs).^1^ At present, the mainstream therapy is chemotherapy, which does not prevent the patient from AML relapse. The 5‐year overall survival (OS) of patients younger than 60 is 40% and only 10%–20% for those aged 60 years and older.^2^ The European LeukemiaNet (ELN) integrated cytogenetics, and molecular data were implemented to perform the risk stratification at diagnosis.^3^ Although the ELN 2017 system has been extensively applied as a tool to evaluate AML patients’ prognosis, due to the complexity of AML, the results of this stratification are still not perfect. Hence, further studies of critical potential biomarkers related to the prognosis of AML are needed.

Rapid progress in sequencing technology nowadays has bought noncoding RNA (ncRNA) to light, exceptionally long non‐coding RNAs (lncRNAs), which have critical roles in malignant transformation and progression.^4^ LncRNAs are participators in regulating gene expression through chromatin modification and the process of transcriptional and posttranscriptional.^5^ As more and more studies on lncRNA function have been revealed, lncRNAs can regulate biologic processes and serve as a prognostic biomarker in many solid tumors.^6^, ^7^, ^8^, ^9^, ^10^ Moreover, lncRNAs also regulate the hemopoiesis system.^11^ For example, HOTTIP is abnormally activated in AML, and HOTTIP loss leads to the inhibition of genes crucial for hematopoiesis and AML leukemogenesis.^12^ Knockdown of LncRNA ANRIL results in a drop in glucose uptake and blockage of AML cell maintenance.^13^ In addition, lncRNA USP30‐AS1 is highly expressed in AML and promotes the survival of acute myeloid leukemia cells by cis‐regulating USP30 and ANKRD13A.^14^

In‐depth research on the cancer‐immune field has led to researchers’ attention on the immune microenvironment in the development of AML.^15^ To some extent, immunotherapy was not novel for the long history of successful application of allo‐SCT in AML. Immune checkpoint therapy and chimeric antigen receptors have made significant tumor immunotherapy progress.^16^ However, AML has not benefited from such breakthroughs, mainly due to the lack of actionable immune targets. Research on AML immunotherapy has been lagging far behind solid tumors.^17^ We have much to do to get a better understanding of the AML immune microenvironment.

Previous IRL‐related study explored the immune‐related competing endogenous RNA network and their association with AML prognosis.^18^ Using the ESTIMATE algorithm, they divided AML patients into low‐risk and high‐risk groups based on immune and stromal scores.^19^ Differentially expressed genes including mRNA, miRNA, and lncRNA were identified and these genes were subjected to construct a competing endogenous RNA network. Finally, each gene in the competing endogenous RNA network was evaluated for the association with AML prognosis. They failed to develop a model for predicting AML patients’ prognosis is a regret. In this study, we identified IRLs using Pearson correlation analysis and constructed an IRL‐based prognostic model, filling the gap in AML.

## 2 METHODS

### 2.1 Data collection

RNA‐Seq (FPKM) data and clinical features of AML patients were obtained from the TCGA‐LAML cohort. All samples included in this study were accorded with the inclusive criteria: (a) newly diagnosed AML samples and (b) availability of transcriptome and clinical data. AML patients were randomly divided into training and test cohorts (ratio 1:1) for building and validating the IRL model, respectively. Computer‐generated random number sequences performed randomization. The clinicopathologic features of AML patients are detailed in Table 1. Immune‐related genes (IRGs) were acquired from the ImmPort database (https://immport.niaid.nih.gov) and 1731 genes were extracted after integrating with the mRNA data from the TCGA‐LAML cohort.

**TABLE 1:** Correlation between clinicopathologic characteristics and the immune‐related lncRNA signature in the TCGA‐LAML cohort

Variables	Training cohort (*n* = 76)			Testing cohort (*n* = 75)			Entire TCGA (*n* = 151)		
	High risk	Low risk	*P*	High risk	Low risk	*P*	High risk	Low risk	*P*
Age (years)									
<65	21	34	**0.002**	23	29	**0.018**	44	63	**0.000**
≥65	17	4		17	6		34	10	
Gender									
Female	13	19	0.163	15	21	0.052	28	40	**0.020**
Male	25	19		25	14		50	33	
Status									
Alive	6	26	**0.000**	5	17	**0.001**	11	43	**0.000**
Dead	32	12		35	18		67	30	
WBC									
<10 × 10^9^/L	16	12	0.342	14	16	0.345	30	28	0.989
≥10 × 10^9^/L	22	26		26	19		48	45	
BM blast									
<70%	23	18	0.179	13	12	0.870	36	30	0.531
≥70%	15	20		27	23		42	43	
ELN2017									
Favorable	4	13	**0.002**	6	9	**0.088**	10	22	**0.000**
Intermediate	12	14		16	9		28	23	
Adverse	22	7		18	6		40	13	
Transplant									
Yes	13	22	**0.038**	20	12	0.170	33	34	0.598
No	25	16		20	23		45	39	
Chemotherapy									
Yes	35	38	0.240	38	35	0.495	73	73	0.059
No	3	0		2	0		5	0	
Relapse									
Yes	14	17	0.674	20	16	0.897	34	33	0.723
No	24	21		20	17		44	38	

### 2.2 Establishment of the IRL signature

Pearson correlation analysis was conducted to identify IRLs between IRGs and lncRNAs from the TCGA‐LAML cohort, and the selection criteria were set to |*R*| >0.8 and *p* value <0.001. At last, we obtained 70 IRLs. To evaluate the association between IRLs and OS, univariate Cox regression analysis was conducted, and nine of them had prognostic values (Table S1). LASSO regression analysis was used to minimize the risk of overfitting, and a multiple stepwise Cox regression method was applied to identify hub IRLs for establishing the prognostic model. The risk score was calculated using the following equation: β1 × gene1 expression + β2 × gene2 expression + β3 × gene3 expression … + β*n *× gene*n* expression, where β was the correlation coefficient produced by the multiple Cox regression analysis.

### 2.3 Evaluation of the IRL signature

Based on the risk score median, AML patients were classified into high‐risk and low‐risk groups. Next, the Kaplan‐Meier analysis was performed to assess the IRL signature's prognostic value. The sensitivity and specificity of the IRL signature were assessed by the receiver operating characteristic (ROC) curve. In addition, univariate and multivariate Cox analyses were applied to prove the model was an independent prognostic model. A prognostic nomogram was then established to predict 1‐, 2‐, and 3‐year OS of AML patients. The calibration curve was plotted to evaluate the accuracy of this nomogram.

### 2.4 Refinement of the European LeukemiaNet (ELN) 2017 System

Patients were divided into the three novel groups: ELN favorable/IRL^high^ and ELN adverse/IRL^low^ patients were reclassified to the intermediate‐risk group. ELN intermediate/IRL^high^ patients were reclassified to the high‐risk group. ELN intermediate/IRL^low^ patients were reclassified to the low‐risk group. We assessed the new risk stratification system's prognostic significance using Kaplan–Meier analysis.

### 2.5 Immune cell‐infiltrating and GSEA analyses among risk groups

CIBERSORT package was applied to explore the differences in several immune cell subtypes among risk groups. The Mann‐Whitney *U* test was used to identify differences among risk groups. ESTIMATE algorithm was applied to study the status of infiltrating immune cells among two subgroups. Gene set enrichment analysis (GSEA) performed by GSEA software was applied to evaluate all gene functions associated with risk groups.

### 2.6 Statistical analysis

The R software (version 4.0.2, https://www.r‐project.org/) was used to perform all statistical analyses. “survival” package was applied to perform the univariate and multivariate Cox regression analyses. “glmnet” and “survival” packages were applied to conduct LASSO regression analysis. Riskscore curve and survival scatter diagrams were performed using the “pheatmap” package. Kaplan–Meier analysis was conducted by “survminer” and “survival” packages. “survivalROC” package was used to determine area under the curve (AUC) values and construct ROC curves. The “rms” R package constructed nomogram. All statistical tests were two‐sided, and *p *< 0.05 was considered to be statistically significant.

## 3 RESULTS

### 3.1 Identification of IRL and construction of the prognostic model in AML patients

**FIGURE 1:**
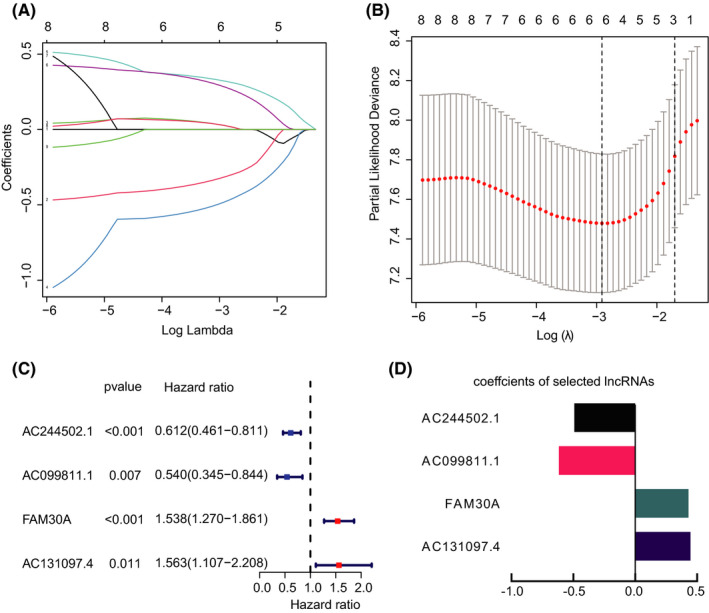
Establishment of the IRL model. (A‐B) LASSO regression was performed to calculate the minimum criteria. (C) Forest plot of the four IRLs included in the prognostic signature. (D) The coefficients of four lncRNAs were estimated by multivariate Cox regression

To construct the prognostic model of AML, 76 samples data sourced from TCGA served as the training set. The mRNA data from the TCGA database and the immune‐related gene set from the ImmPort database were intersected to obtain 1731 immune genes related to AML. Pearson correlation analysis between lncRNA expression and 1731 immune‐related gene expression identified nine IRLs. These nine prognostic IRLs were subjected to LASSO regression analysis and six IRLs were selected, including AC244502.1, AC025259.3, AC099811.1, FAM30A, AC131097.4, and U62631.1 (Figure 1A,B). Multiple stepwise Cox regression was conducted to select the IRLs with better prognostic value, and four hub IRLs were produced to construct the prognostic model for AML patients (Figure 1C). The hazard ratio of AC244502.1 and AC099811.1 is <1. The hazard ratio of FAM30A and AC131097 is >1. The coefficient for calculating the risk score is shown in Figure 1D.

### 3.2 Evaluation of the prognostic model

**FIGURE 2:**
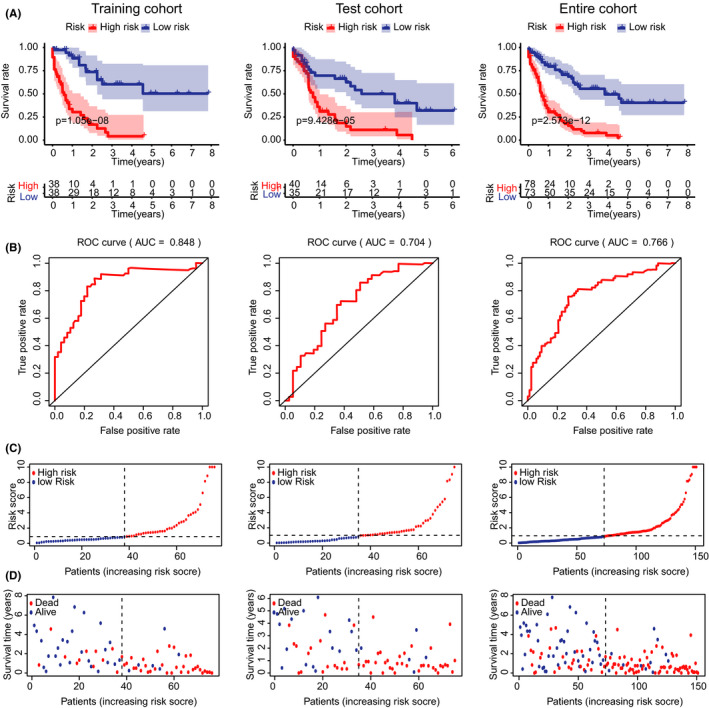
Evaluation of the IRL model. (A) Kaplan–Meier analysis for the subgroups in the TCGA‐LAML cohort. (B) ROC curves of IRLs for predicting the survival in the subgroups. (C‐D) The distribution of risk scores and survival status of AML patients in the subgroups

With the IRL signature, we divided the patients into high‐risk and low‐risk groups. Kaplan–Meier survival curves depicted that AML patients with higher risk scores had worse clinical outcomes in the test, training, and entire cohorts, respectively (Figure 2A). The ROC curves indicated that the IRL model could accurately predict OS in the TCGA cohort (training cohort AUC = 0.848, test cohort AUC = 0.704, entire cohort OS = 0.766; Figure 2B). Survival status distributions and risk scores are plotted in Figure 2C,D. We observed that patients in high‐risk groups had a higher mortality rate. Through the above analysis, the prognosis model we constructed showed a convincing judgment on the prognosis of AML patients.

### 3.3 Stratification analysis

**FIGURE 3:**
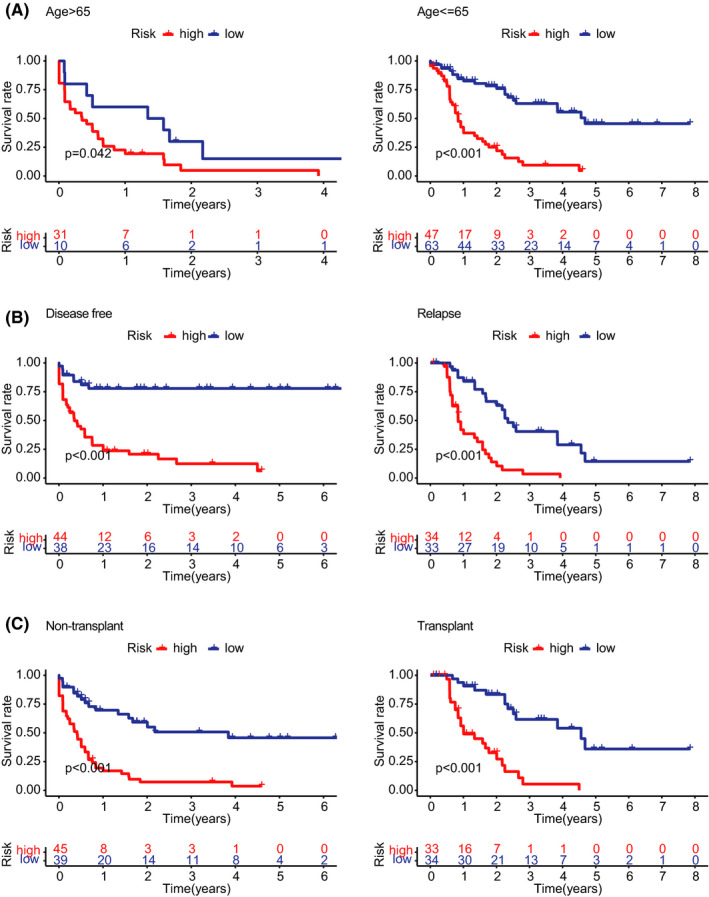
Stratification analysis. (A) Kaplan–Meier analysis of the IRL model in patients aged ≤40 or >40 years groups. (B) Kaplan–Meier analysis of the IRL model in disease‐free or relapse groups. (C) Kaplan–Meier analysis of the IRL model in nontransplant or transplant groups

We intended to ascertain whether the IRL signature could be applied to different clinicopathologic subgroups. In the subgroup of age ≤65, the prognostic model retained its predictive power of telling the OS (Figure 3A). However, in the senior age group, the difference in OS between the low‐risk and the high‐risk groups is not as distinct as in the age ≤65 (Figure 3A). It implied that old age has a massive impact on prognosis. Likewise, compared with patients with lower risk, AML patients with higher risk had worse OS in the relapse or disease‐free subgroups (Figure 3B). We also confirmed that the IRL model could still accurately predict OS for patients who have undergone a transplant or not (Figure 3C). According to different clinical information to stratify patients, our model still shows good prognostic prediction ability.

### 3.4 The IRL prognostic model was an independent factor for AML patients’ prognosis

**FIGURE 4:**
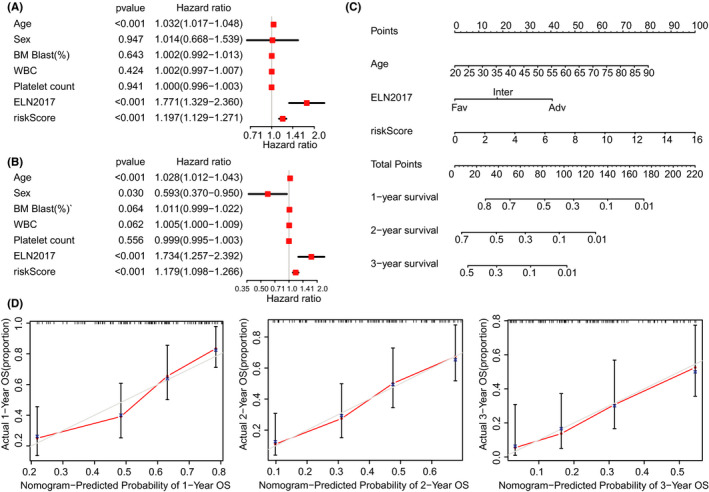
Establishment and evaluation of the nomogram. (A) Univariate analyses of the IRL model. (B) Multivariate analyses of the IRL model. (C) Nomogram based on IRL model's risk score and clinical information. (D) Calibration plots for predicting the probability of OS at 1, 2, and 3 years

We applied the univariate and multivariate Cox analyses to evaluate whether the IRL prognostic model was an independent factor for AML patients’ prognosis. The univariate Cox analysis indicated that the IRL prognostic model was significantly associated with OS (hazard ratio = 1.197, *p* < 0.001; Figure 4A). The multivariate Cox analysis further displayed that the IRL prognostic model was an independent predictor of OS (HR: 1.179, *p* < 0.001; Figure 4B).

### 3.5 Construction and evaluation of nomogram

Integrated with conventional reliable risk factors, we can establish a nomogram containing the IRL signature to predict AML patinets’ prognosis quantitatively. The risk status (based on the IRL prognostic model), ELN2017 risk stratification system, and age in the TCGA data set were used to construct a nomogram (Figure 4C). The calibration plots indicated that the observed vs. predicted rates of 1‐, 2‐, and 3‐year OS had an excellent concordance in the TCGA (Figure 4D). These results demonstrated that the nomogram had an excellent ability to predict AML patients’ prognosis.

### 3.6 Incorporation with the IRL prognostic model improved the ELN2017 risk stratification system

**FIGURE 5:**
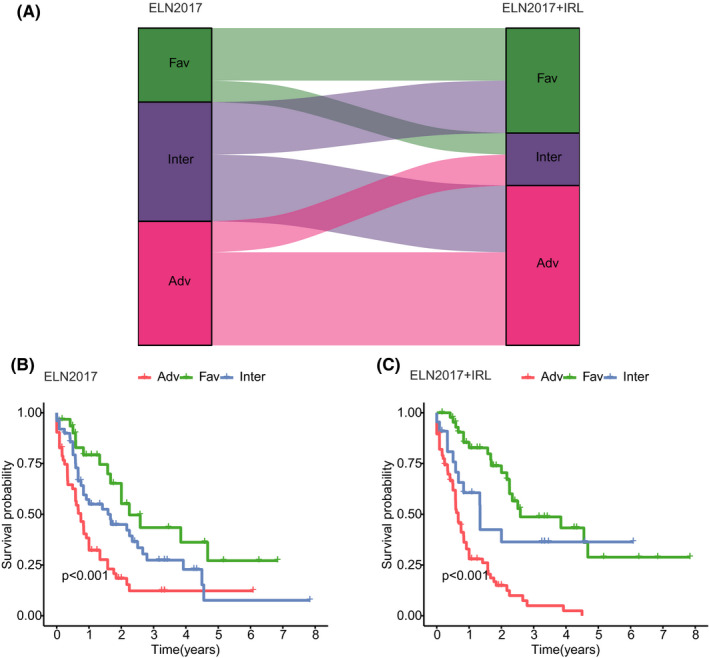
Improved ELN2017 system. (A) Reclassification of patients according to incorporating the ELN2017 risk stratification system and the IRL model. (B) Kaplan–Meier analysis for AML patients classified by ELN2017 system or (C) ELN2017‐IRL model system

ELN2017 risk stratification system is widely used for AML patients’ risk stratification. However, the prognostic heterogeneity calls for a refinement of the risk stratification. We then compared our ELN2017+ IRL model with ELN2017. The results indicated that 29% of patients in the favorable group and 25% in the adverse group of ELN2017 were reclassified into the adverse group of our model, 44% of patients in the intermediate group of ELN2017 were re‐classified into the favorable group and 56% of patients in the intermediate group of ELN2017 were reclassified into the adverse group (Figure 5A). In addition, we found that the combined ELN‐IRL risk stratification system could more accurately define AML patients’ prognosis (Figure 5B,C).

### 3.7 Correlation between risk score and status of tumor‐infiltrating immune cell

**FIGURE 6:**
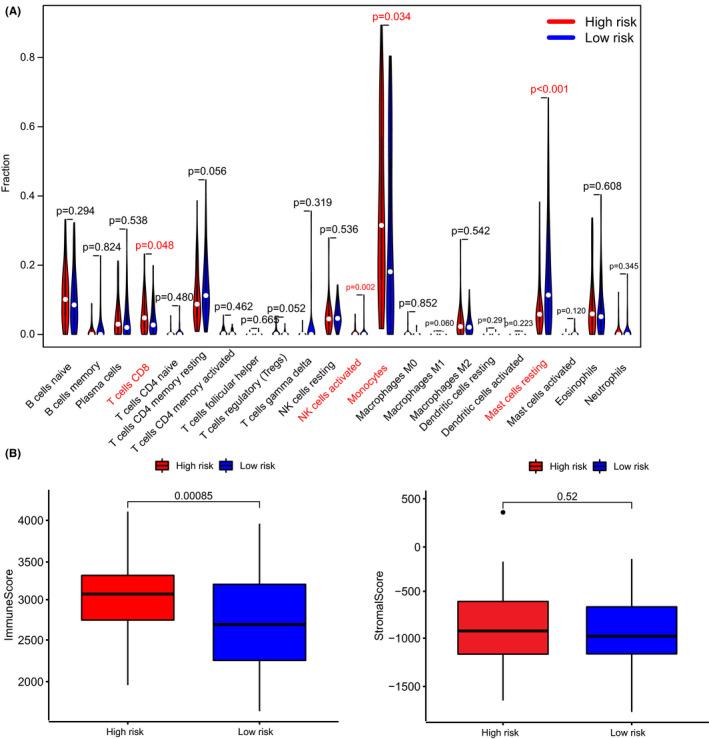
Association between risk score and immunity. (A) Violin plot of the 22 infiltrating immune cells. (B) The results of the ESTIMATE algorithm in the high‐risk and the low‐risk groups

Immune cells play diverse roles in tumor development and eradication. The 22 different immune cell types among low‐ and high‐risk groups were analyzed with the CIBERSORT algorithm. We can see in Figure 6A that the high‐risk group displayed a more significant number of CD8^+^ T cells, activated NK cells, and monocytes. Also, we found that the levels of mast cells resting in the high‐risk group were significantly lower than those in the low‐risk group. Figure 6B shows that the immune score in the high‐risk group is higher than it in the low‐risk group. Moreover, the stroma score has no difference between the two groups.

### 3.8 GSEA analysis

**FIGURE 7:**
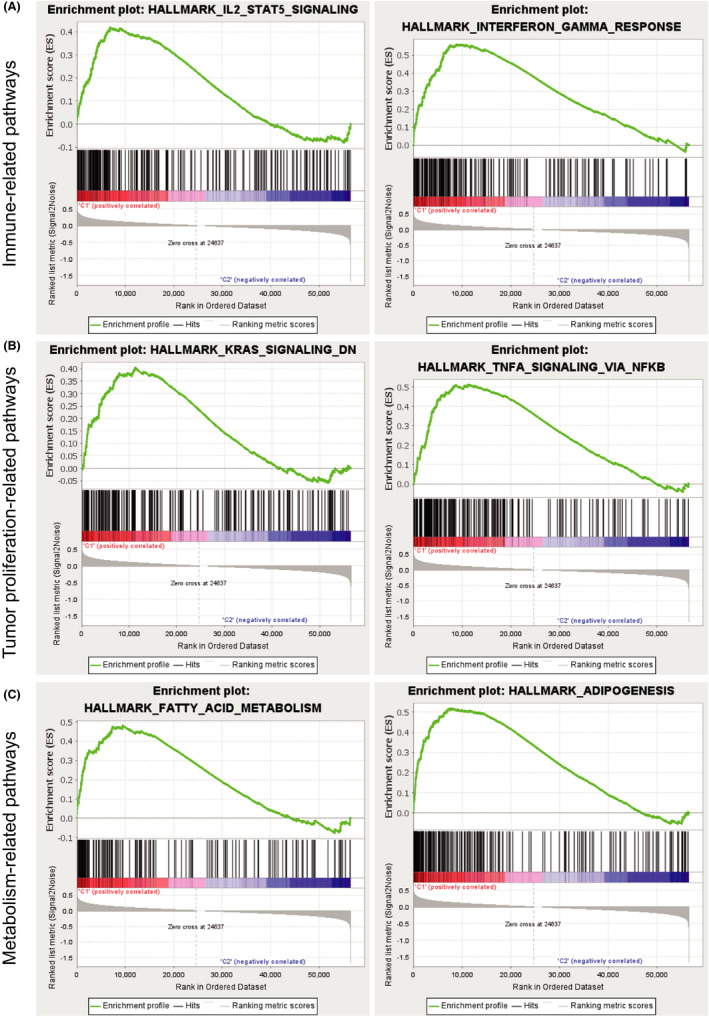
GSEA analysis. (A) Immune‐related pathways. (B) Tumor proliferation‐related pathways. (C) Metabolism‐related pathways

To explore the potential biologic differences between high‐ and low‐risk groups, we performed a GSEA analysis. These pathways could be roughly divided into three categories: immune‐related (Figure 7A), tumor proliferation‐related (Figure 7B), and metabolism‐related pathways (Figure 7C). The hallmark pathways enriched in high‐risk patients are IL2‐STAT5 signaling, IFN‐γ response, TNF‐α‐NFκB signaling, k‐RAS signaling, adipogenesis, and fatty acid metabolism. These data informed us to explore the mechanism further in future work.

## 4 DISCUSSION

Patients with identical cytogenetic and molecular “makeup” may not have uniform outcomes.^20^, ^21^ These inconsistent prognostic outcomes may result from additional undiscovered critical factors. LncRNAs are crucial regulators of gene expression and play a significant part in cancer physiology and pathology.^6^, ^22^ This study constructed a novel and reliable IRL model to predict AML patients’ OS and improved the ELN2017 risk stratification system's accuracy.

There are few reports on these four immune‐related lncRNAs, and especially AC244502.1, AC099811.1, and AC131097.4 are considered as novel lncRNAs. AC244502.1 is reported to be a protective factor (HR = 0.777) in breast cancer.^23^ AC131097.4 is a risk factor (HR = 2.019) for low‐grade glioma.^24^ FAM30A is highly expressed in B cells and plays an important role in immune regulation, including inflammatory immune response accompanied with B cells activation^25^ and various vaccine response‐related immune pathways.^26^ In addition, FAM30A is reported to be a biomarker in many types of tumors, like gastric cancer,^27^ lung adenocarcinoma,^28^ laryngeal squamous cell carcinoma,^29^ and AML.^30^

Nomograms are helpful and reliable tools for physicians to plan individualized treatment, predict survival, and decide the interval for follow‐up and/or imaging.^31^ In this study, we found that these variables, including WBC, platelet, sex, and BM Blast, were not statistically significant in the univariate and multivariate Cox analyses and dropped from the model. In contrast, age, ELN2017 stratification, and riskScore variables were significant in both analyses and integrated to form a nomogram, which provided an individualized survival estimate.

ELN2017 classification, integrated cytogenetic and mutational status information, is widely used in clinical practice.^3^ However, remarkable heterogeneity remains unresolved. Because immune‐related factors were not included in this classification, we integrated our IRL model with the ELN2017 classification, and this fused model had a better prediction efficiency than the ELN2017 classification only.

Our study showed that the high‐risk group displayed more CD8^+^ T cells, monocyte infiltration, and more immune components. The GSEA analysis illustrated that the IFN‐γ response was enriched in the high‐risk group. As CD8^+^ T cells directly kill tumor cells and IFN‐γ acts as a proapoptotic signal for tumor cells,^32^, ^33^ the phenomenon that these tumor‐suppressive factors are enriched in the high‐risk group seems to be contrary to our cognition. However, IL2‐STAT5 signaling is also highly expressed in the high‐risk group. Recent studies show that a high level of IL‐2 induces a persistent STAT5 activation in CD8^+^ T cells,^34^ and the IL2‐STAT5 pathway is positively related to the CD8^+^ T cell exhaustion and Treg cells maintaining.^35^, ^36^ Also, differentiated monocyte‐like AML cells have immunosuppressive functions that contribute to the pathogenesis of this disease.^37^ Hence, we assume that although antitumor immune‐related components are more in the high‐risk group, their immune exhaustion and suppression are more severe. So the original antitumor effect is diminished. Moreover, the TNF‐α‐NFκB signaling and k‐RAS signaling pathways are prone to promote cancer proliferation. We guess that in the TME of the high‐risk group, the anti‐tumor immune system lost to tumor proliferation signaling pathways.

Furthermore, adipogenesis and fatty acid metabolism are enriched in the high‐risk group. High serum‐saturated fatty acids can cause inflammation, and lipid metabolism disorder is vital in cancer advancement.^38^ De novo fatty acid (FA) synthesis is indispensable for T effector cell differentiation, and CD8^+^ T memory cell behavior relies on FA synthesis and oxidation.^39^ Our GSEA analysis indicated that metabolism plays a vital role in AML progression.

With the development of bioinformatics and sequencing technology, many AML prognosis prediction models are established. The abnormal expression of RNA‐binding proteins (RBPs) plays a role in multiple cancers. The 12‐RBP prognostic signature was established, which can effectively stratify the risk of AML patients.^40^ Hypoxia can activate a series of immunosuppressive processes in tumors, resulting in a poor clinical prognosis. A hypoxia risk signature was developed to predict clinical prognosis.^41^ Our previous study constructed an immune‐related gene signature to clarify the important role of immune factors and contribute to the improved prediction of AML prognosis.^42^ This study used Pearson correlation analysis to identify IRL genes for the first time in AML and constructed an IRL signature, which revealed a good prediction efficiency. We also identified three novel lncRNAs that may play important role in the development of AML. To a certain extent, several limitations of this study should be considered. (a) Three are three novel lncRNAs in our signature that are not reported in the previous study, which need to be studied using biologic experiments, (b) the number of samples in this study is limited and more patients need to be included in the future, (c) AML is a multifactorial disease and complex interactions between genetic and environmental factors. The investigation of single immune factors cannot interpret the association of AML risk comprehensively.

In conclusion, we established a reliable IRL signature to predict AML patients’ OS and improved our prognostic model's ELN2017 risk stratification system. In the future, more high‐quality clinical studies are needed to confirm this model's accuracy and applicability.

## CONFLICT OF INTEREST

The authors declare no conflict of interest.

## AUTHORS’ CONTRIBUTIONS

Ran Li and Junmin Li designed this work. Ran Li, Shishuang Wu, and Xiaolu Wu integrated and analyzed the data. Ran Li and Kai Xue wrote this manuscript. Ran Li, Shishuang Wu, and Xiaolu Wu edited and revised the manuscript. All authors approved this manuscript.

## ETHICS STATEMENT

Not applicable.

## Supporting information

Table S1

## Data Availability

RNA‐Seq (FPKM) data and clinical information of AML patients were downloaded from the TCGA‐LAML cohort (https://portal.gdc.cancer.gov/).
