# Supplementary material for: Immune‐relatedlncRNAs can predict the prognosis of acute myeloid leukemia
Source: Cancer Med. 2021 Dec 14;11(3):888–99. doi: 10.1002/cam4.4487 (PMC8817083; doi:10.1002/cam4.4487)
Supplement: Supplementary file 1 — Table S1 [file CAM4-11-888-s001.docx]

| Table S1. Univariate Cox regression analysis for identified immune-related lncRNAs | | | | |
| --- | --- | --- | --- | --- |
| id | HR | HR.95L | HR.95H | pvalue |
| U62317.2 | 0.91485 | 0.6028 | 1.38843 | 0.67587 |
| AC010889.1 | 0.90659 | 0.73964 | 1.11122 | 0.34498 |
| AC098869.2 | 0.87774 | 0.6509 | 1.18362 | 0.39262 |
| AC007728.3 | 0.83918 | 0.63151 | 1.11516 | 0.22681 |
| AC087623.3 | 0.60036 | 0.29867 | 1.20678 | 0.15205 |
| LUCAT1 | 0.94805 | 0.64352 | 1.39671 | 0.78729 |
| AC006157.1 | 0.73527 | 0.45284 | 1.19386 | 0.2137 |
| AC007278.2 | 1.09875 | 0.7925 | 1.52333 | 0.57214 |
| AC116158.2 | 1.01863 | 0.61101 | 1.6982 | 0.94357 |
| TTTY14 | 0.93266 | 0.73355 | 1.18582 | 0.56939 |
| AC244502.3 | 0.79659 | 0.66334 | 0.95661 | 0.01489 |
| HLA-DQB1-AS1 | 0.97278 | 0.79737 | 1.18679 | 0.78564 |
| SOS1-IT1 | 0.90086 | 0.5724 | 1.4178 | 0.65183 |
| LINC01891 | 1.03928 | 0.56533 | 1.91057 | 0.9013 |
| AC073655.2 | 1.36375 | 0.90366 | 2.05809 | 0.13954 |
| AC078883.1 | 0.93672 | 0.55809 | 1.57225 | 0.80461 |
| Z83843.1 | 0.79812 | 0.56525 | 1.12693 | 0.20016 |
| AC020594.1 | 0.72438 | 0.46638 | 1.12511 | 0.15122 |
| AC244502.1 | 0.78997 | 0.64619 | 0.96575 | 0.02145 |
| LINC00968 | 0.84743 | 0.476 | 1.5087 | 0.57376 |
| VCAN-AS1 | 0.86855 | 0.70782 | 1.06579 | 0.1771 |
| AL109767.1 | 0.75797 | 0.47096 | 1.21987 | 0.25371 |
| AC025259.3 | 1.49911 | 1.04407 | 2.15246 | 0.02826 |
| AC037198.2 | 1.22105 | 0.94695 | 1.57449 | 0.12364 |
| AC138207.5 | 0.89286 | 0.70871 | 1.12485 | 0.33622 |
| AC099811.1 | 0.64126 | 0.43577 | 0.94366 | 0.02419 |
| AKT3-IT1 | 1.10265 | 0.83112 | 1.4629 | 0.4981 |
| FAM30A | 1.34704 | 1.13159 | 1.60351 | 0.00081 |
| AC064805.1 | 0.83631 | 0.62053 | 1.12711 | 0.24036 |
| AC244035.1 | 0.66126 | 0.39335 | 1.11163 | 0.1186 |
| AC008753.2 | 1.0469 | 0.83746 | 1.30873 | 0.68734 |
| C1RL-AS1 | 1.34756 | 0.78316 | 2.31871 | 0.28137 |
| AL355922.1 | 0.86201 | 0.73416 | 1.01213 | 0.06987 |
| AL359962.2 | 1.17161 | 0.84432 | 1.62577 | 0.34337 |
| AC004906.1 | 1.45433 | 0.87803 | 2.4089 | 0.14574 |
| AC087623.1 | 0.84418 | 0.59099 | 1.20585 | 0.35182 |
| XIST | 1.00096 | 0.90969 | 1.10138 | 0.98436 |
| AC097103.1 | 0.60002 | 0.34888 | 1.03196 | 0.06486 |
| AC138207.1 | 0.89374 | 0.6072 | 1.31551 | 0.56897 |
| AC123567.2 | 1.18512 | 0.83875 | 1.67453 | 0.33557 |
| AC010609.1 | 1.13451 | 0.7363 | 1.74809 | 0.56722 |
| PRKCQ-AS1 | 1.29748 | 0.81147 | 2.07457 | 0.27679 |
| AC131097.4 | 1.37862 | 1.05069 | 1.80891 | 0.02052 |
| AC093642.1 | 0.96307 | 0.54415 | 1.7045 | 0.89721 |
| AC073655.1 | 1.28429 | 0.92048 | 1.79189 | 0.14093 |
| AC069029.1 | 0.69116 | 0.419 | 1.14012 | 0.14805 |
| AL359962.1 | 1.13448 | 0.83535 | 1.54071 | 0.41912 |
| AC008753.3 | 1.06134 | 0.83287 | 1.35249 | 0.63025 |
| AP001257.1 | 0.94864 | 0.71517 | 1.25832 | 0.7145 |
| AC098613.1 | 0.99078 | 0.68074 | 1.44202 | 0.96142 |
| AC116348.2 | 1.00233 | 0.67683 | 1.48438 | 0.99072 |
| AC037459.2 | 0.76631 | 0.41235 | 1.42408 | 0.39988 |
| AC126614.1 | 0.89567 | 0.59375 | 1.35112 | 0.59939 |
| AC037198.1 | 1.23627 | 0.93842 | 1.62866 | 0.13154 |
| TTTY10 | 1.09226 | 0.77292 | 1.54355 | 0.61697 |
| AC119428.2 | 0.8387 | 0.57948 | 1.21387 | 0.35107 |
| SMIM25 | 0.98545 | 0.82189 | 1.18156 | 0.87426 |
| AC018653.3 | 1.27703 | 0.83285 | 1.95811 | 0.26217 |
| TRG-AS1 | 0.94585 | 0.6401 | 1.39763 | 0.77988 |
| AC007278.1 | 1.14693 | 0.88491 | 1.48652 | 0.30021 |
| AC099811.5 | 0.6746 | 0.45619 | 0.99755 | 0.04858 |
| AC025917.1 | 0.65318 | 0.38101 | 1.11977 | 0.12147 |
| AC245128.3 | 1.00987 | 0.85929 | 1.18684 | 0.9051 |
| U62631.1 | 1.59611 | 1.00798 | 2.52741 | 0.04617 |
| AL591721.1 | 1.27589 | 0.85512 | 1.90371 | 0.23272 |
| PRKCA-AS1 | 0.75513 | 0.48944 | 1.16505 | 0.20427 |
| AC015911.3 | 1.18147 | 0.84531 | 1.65131 | 0.32896 |
| AC246787.2 | 1.22765 | 1.04546 | 1.4416 | 0.01234 |
| TRBV11-2 | 1.26607 | 0.69892 | 2.29347 | 0.43642 |
| AC006947.1 | 0.64422 | 0.39784 | 1.04318 | 0.07376 |
